# Classroom teaching versus online teaching in physiology practical course – does this lead to different examination results?

**DOI:** 10.3205/zma001732

**Published:** 2025-02-17

**Authors:** Tom Dreyer, Symeon Papadopoulos, Rudolf Wiesner, Yassin Karay

**Affiliations:** 1 University of Cologne, Faculty of Medicine, Cologne, Germany; 2 University of Cologne, Faculty of Medicine, Institute of Neurophysiology, Cologne, Germany; 3 University of Cologne, Faculty of Medicine, Institute for Systems Physiology, Cologne, Germany; 4 University of Cologne, Faculty of Medicine, Dean’s Office, Cologne, Germany

**Keywords:** medical teaching, physiology, online teaching, face-to-face teaching, examination results

## Abstract

**Background:**

Due to contact restrictions during the Corona pandemic, teaching at the Center for Physiology and Pathophysiology at the University of Cologne was temporarily offered online for some students and face-to-face for others. As there are different views on the effectiveness of online teaching, this study compared students’ examination results between the teaching formats (face-to-face vs. online).

**Methods:**

In winter 2021/22, a total of 198 students in their fourth preclinical semester took part in the physiology course. The students were randomly assigned to 15 practical courses, so that the practical course was completed either traditionally in presence (face-to-face; FtF_group_) or as an online practical course via Zoom^®^ (O_group_). The teaching format versus the score achieved per test question were recorded for each examinee. The differences in test scores were calculated using a two-sided t-test. The effect size was determined using Cohen’s d. Spearman’s rank correlation coefficient was used as a measure of the correlation.

**Results:**

In comparison with the O_group_ (M=7.02), the FtF_group _(M=7.38) achieved a significantly higher test score on average. The effect size was low (Cohen’s d=0.135). The FtF_group_ performed better than the O_group_ in 14 subject areas. The Spearman’s correlation test between the number of FtF participations and the test scores achieved reached a value of r=0.236 (p<.001).

**Conclusion:**

Our study shows that students who have attended traditional face-to-face classes tend to perform better in the written exam. The reasons may be multifactorial. However, online teaching also offers some advantages, such as flexibility in terms of location and time management for students. The choice between online and face-to-face teaching should be based on the specific requirements of the course. Ideally, a hybrid solution that combines the advantages of both formats would be an effective teaching format. It is therefore essential to continuously review educational practices.

## Zusammenfassung

**Hintergrund:**

Aufgrund der Kontaktbeschränkungen während der Corona-Pandemie wurde die Lehre am Zentrum für Physiologie und Pathophysiologie der Universität zu Köln zeitweise für einen Teil der Studierenden online und für den anderen Teil der Studierenden in Präsenz angeboten. Da es verschiedene Auffassungen zur Wirksamkeit von Online-Lehre gibt, wurden in dieser Studie die Prüfungsergebnisse der Studierenden zwischen den Unterrichtsformaten (Präsenz vs. Online) verglichen.

**Methoden:**

Im Wintersemester 2021/22 haben insgesamt 198 Studierende des vierten vorklinischen Semesters am Kurs Physiologie teilgenommen. Die Studierenden wurden auf 15 Praktika randomisiert aufgeteilt, so dass das Praktikum entweder klassisch in Präsenz (Face-to-face; FtF_group_) oder als Online-Praktikum via Zoom^®^ (O_group_) absolviert wurde. Je Prüfling wurde das Unterrichtsformat und die erreichte Punktzahl pro Frage der Prüfung erfasst. Die Unterschiede beim Testscore wurden mit Hilfe des zweiseitigen t-Tests berechnet. Die Effektstärke wurde mit Cohen’s d ermittelt. Als Maß des Zusammenhangs wurde der Spearman’sche Rangkorrelationskoeffizient verwendet.

**Ergebnisse:**

Im Vergleich mit der O_group_ (M=7,02) erzielte die FtF_group_ (M=7,38) im Durchschnitt einen signifikant höheren Testscore. Die Effektstärke war gering (Cohens’d=0,135). Bei 14 Themengebieten schloss die Face-to-Face-Gruppe besser ab als die Online-Gruppe. Der Korrelationstest nach Spearman zwischen Anzahl an FtF-Teilnahmen und den erzielten Testpunkten erreicht einen Wert von r=0,236 (p<.001).

**Schlussfolgerung:**

Unsere Studie zeigt, dass Studierende, die klassischen Präsenzunterricht absolviert haben, tendenziell besser bei der schriftlichen Klausur abschneiden. Dies kann multifaktoriell bedingt sein. Jedoch bietet auch der Online-Unterricht einige Vorteile, wie z.B. örtliche und zeitliche Flexibilität für die Studierenden. Die Wahl zwischen einem Online- und einem Präsenzpraktikum sollte auf den spezifischen Anforderungen des jeweiligen Kurses basieren. Idealerweise würde eine hybride Lösung, die die Vorteile beider Formate kombiniert, ein effektives Unterrichtsformat darstellen. Daher ist es unumgänglich, Bildungspraktiken kontinuierlich zu überprüfen.

## Introduction

The educational landscape has undergone a remarkable transformation in recent years, driven primarily by the rapid development of digital technologies and their integration into everyday teaching practice. Online teaching in particular has become an integral part of the modern education system. This paradigm shift has been accelerated by various factors such as globalization, the availability of high-speed internet and the COVID-19 pandemic [1], [2], [3]. Online teaching refers to any type of distance learning where the learners are not in the same room as the teachers [4]. This can be done via various platforms and tools such as video conferencing, e-learning systems or webinars.

During the COVID-19 pandemic, the Center for Physiology and Pathophysiology at the University of Cologne was also faced with the challenge of offering its practical classes online synchronously via Zoom^®^ in addition to the face-to-face format, in order to comply with the legal contact restrictions in force at that time. Due to restrictions in the maximum number of people per classroom, not all students were able to complete all practical classes in person. Students were therefore randomly assigned to either online or face-to-face lessons.

Online teaching can offer some advantages such as flexibility, accessibility and time saving. However, there are also concerns about the suitability of the online format in terms of the content to be taught. Some studies have highlighted important challenges such as lack of social interaction, distractions at home and the need for self-discipline [5]. These factors could therefore indirectly influence the students’ examination performance. It is known that different teaching formats can have different effects on learning. For example, studies have shown that students often perform better in traditional, interactive teaching formats such as seminars, group work or discussion groups than in traditional lectures, as they can actively influence their own learning process [6]. However, there are only a few studies to date that deal specifically with the relationship between performance in an exam and the comparison between online and face-to-face teaching in medical education.

In this study, we investigated precisely this question and examined whether there is a correlation between the teaching format (face-to-face vs. online) and performance in the final exam. Our results should help to further optimize the use of online teaching in higher education and thus improve the overall quality of learning.

## Methods

### Educational context

Medical studies in Germany lead to the completion of the state examination and entitle students to practice medicine. The minimum period of study is 12 semesters and 3 months (including exam preparation). At the Medical Faculty of the University of Cologne, the study of human medicine has been offered as a model course of study (pilot study program) since the winter semester 2003/04 [7]. The basis of teaching is systematic instruction in the classical subject areas supplemented by interdisciplinary, practice-oriented, patient-oriented and science-oriented teaching. The Cologne curriculum consists of a first preclinical (2 years) and a second clinical study phase (3 years) as well as a subsequent practical year (1 year). In the first phase, students learn basics of natural sciences and medicine in particular. The subjects include general medicine, biology, physics, chemistry, anatomy, biochemistry, physiology, histology, medical psychology and sociology.

In Cologne, the subject of physiology consists of a series of 85 hours of lectures, practical courses including seminars in small groups and a final examination. In the Cologne curriculum, physiology is taught in the fourth pre-clinical semester. Students have to complete a total of 16 compulsory practical classes including seminars on the following topics: Nerve function, dioptrics/retina, energy metabolism, psychophysiology, electroencephalo-graphy (EEG), motor system/reflexes, pH regulation, cardiac electrophysiology, cardiac mechanics, acoustics/balance, autonomic nervous system (VNS), circulation, pulmonary function, muscle physiology, renal physiology and blood function. Each course lasts 7 hours with typically 1.5 hours of a theoretical introduction, a 45-minute break, followed by topic-specific experiments and a final debriefing and discussion of the results.

The students had to complete an average of 2 to 3 practical classes per week. Written instructions for each topic were provided. The students had the task of writing protocols in which they described and discussed the results of their experiments. In contrast to face-to-face practical courses, students in the online practical courses were obviously not able to carry out practical experiments themselves, which were instead demonstrated by live videos or similar material and explained interactively by teaching staff via video conferences (Zoom^®^). During the practical course, the video conferences were not recorded in order to offer students a learning environment that was as relaxed as possible, similar to the process in the face-to-face practical course. Some of the experiments took place in the face-to-face setting using simulation programs. These programs were made available to the online students by means of a license release. All lectures were held online for everyone via Zoom^®^. These Zoom lectures were held live while being recorded. Recordings were only accessible to the students of the course after being uploaded to the university e-learning system ILIAS^®^ and were accessible until the examination and beyond. The students were informed of the recordings in advance. Only the lecturers were visible and audible in the recordings.

As in previous years, the final exam took place simultaneously for all students in person and lasted 150 minutes. The exam consisted of a total of 40 open questions. A maximum of 4 points could be achieved per question, meaning that a maximum of 160 points could be achieved. The minimum score to pass the exam was 60%=96 points. The exam questions were prepared by the teachers who had supervised the corresponding practical course topics. Each topic was represented by 2 or 3 questions: 2 questions each on nerve, dioptric/retina, energy balance, psychophysiology, EEG, reflexes, pH regulation and cardiac electrics, and 3 questions each on cardiac mechanics, acoustics/balance, VNS, circulation, respiration, muscle, blood and renal physiology. For the specific group of students in this study, Cronbach’s alpha was 0.92 [8]. The pass rate for the final exam was 84% and was in line with the pass rates of the previous semesters. The mean value of the pass rates over the last 7 semesters was 83.7%, with a fluctuation range of no more than +/-5%.

### Sample and randomization

**Figure 1:**
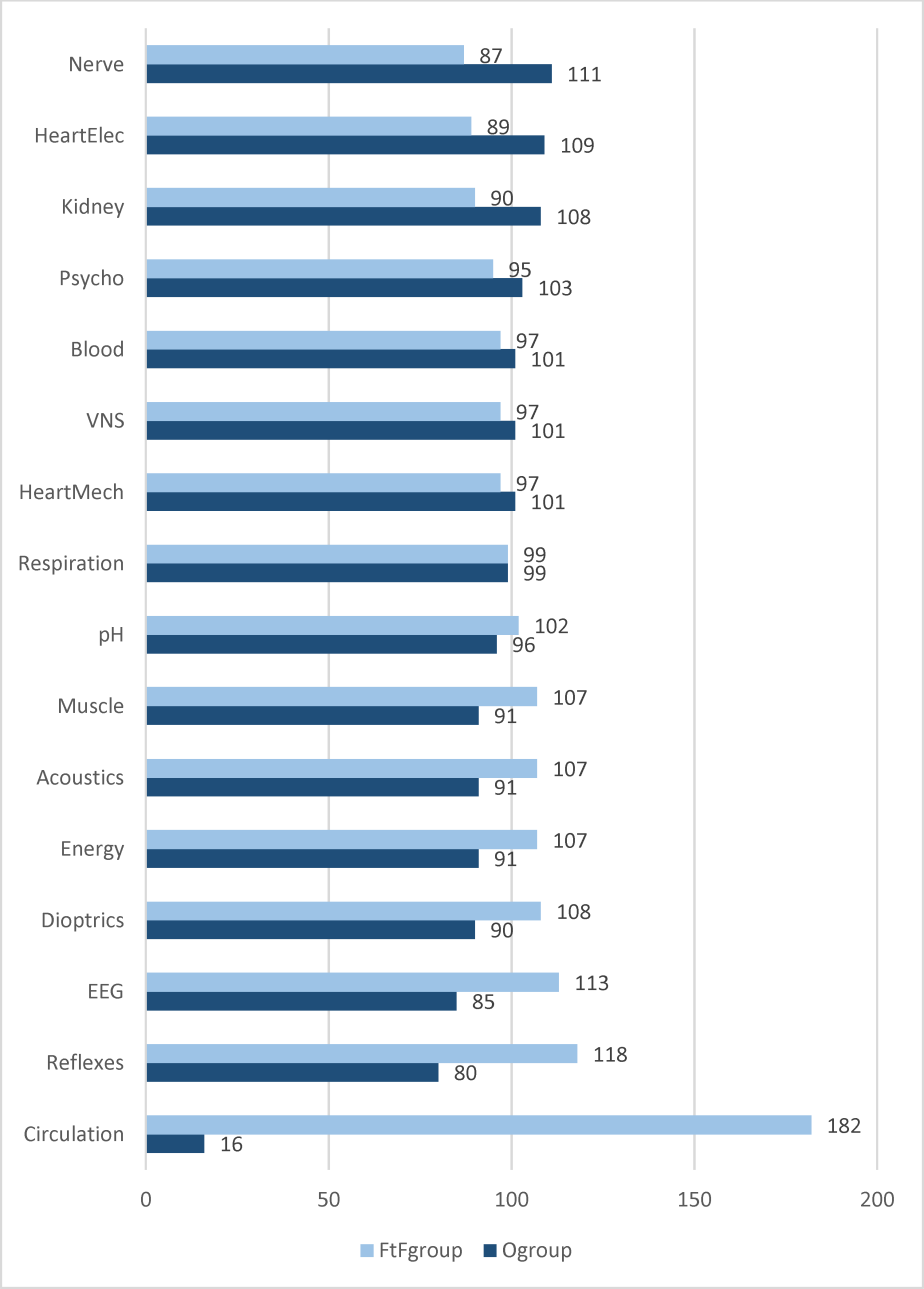
Distribution of students between face-to-face and online teaching

In the winter semester 2021/22, a total of 198 first-time participants in the fourth preclinical semester regularly took part in the physiology course. As this is a compulsory practical course, there were no no-shows. Repetition students, long-term students and drop-outs were not included in the analysis in order to minimize distortions in the results. For 15 of the 16 practical courses, the 198 students were randomly divided into two groups. Students were not randomly assigned to the topic of circulation, but had to appear in person in the form of staggered semi-groups. Nevertheless, 16 students had to complete the “circulation” practical course online because they either had a coronavirus infection or presented a medical certificate exempting them from the obligation to attend face-to-face classes. The randomization for the 15 practical course topics was alternating: The students who were assigned to the online group (O_group_) for a specific topic had to complete the practical course completely online via Zoom^®^, while the students in the face-to-face presence group (FtF_group_) completed the same topic in the institute’s rooms in presence. For the subsequent topic, it was the other way around, etc. It was only possible to deviate from this division if, for example, students were not allowed to take part in face-to-face lessons or could not take part in online lessons due to a coronavirus infection or other serious reasons. On average, students completed 8.6 face-to-face practical classes, with a standard deviation of 1.7. Four students only attended online internships. Two students completed the maximum of 11 face-to-face placements. The distribution of students by teaching format per practical course topic can be seen in figure 1 (Fig. 1).

Only in one case, “respiration”, was the number of students evenly distributed between online and face-to-face seminars. In six of the course seminars, there were more online participants than in the face-to-face courses; in the remaining nine topics, the opposite was true, with the “circulation” practical course representing the extreme of face-to-face teaching with 182 face-to-face participants compared to 16 online participants.

### Statistical analyses

The type of participation in the respective practical course (O_group_ or FtF_group_) and the number of points achieved per question in the exam were recorded for each participant. There were at least 2 and a maximum of 3 test questions for each topic, each of which was awarded a maximum of 4 points (see above for the number of questions per topic). The test score (T_score_) per topic was calculated for each individual student. This study did not analyze exam results by demographic characteristics.

In the first step, we used the two-sided t-test, assuming independent samples, to investigate whether there are differences in the test score between the face-to-face groups (FtF_group_) and the online groups (O_group_). The statistical tests are based on a probability of error of less than 5%, i.e. p-values below 0.05 are considered statistically significant. To quantify the differences between these two groups, we also calculated the effect size based on Cohen’s d. The effect size according to Cohen’s d can take values between -∞ to +∞. The measure can be defined as small |0.2|, medium |0.5| or large |0.8| [9]. In the second step, we used Spearman’s rank correlation coefficient to measure the strength and direction of the correlation between the number of FtF participations and the test scores achieved. The correlation coefficient can assume values between -1 and +1. The correlation can be defined as small |0.1|, medium |0.3|, large |0.5|, very large |0.7| [9]. The sign of the correlation coefficient indicates the direction of the correlation.

## Results

Table 1 (Tab. 1) shows the differences in the test scores (T_score_) achieved per topic and overall between the face-to-face groups (FtF_group_) and the online groups (O_group_). As no randomization took place for the topic “circulation”, the results for the topic “circulation” are only presented for the sake of completeness and neglected in the subsequent discussion. Accordingly, the overall results are presented once without and once with the inclusion of the topic of “circulation”.

The FtF_group_ scored better than the online group on 14 topics. The differences for the topics nerve, pH-Regulation and muscle were significant at the 5% level, each with a small effect size. The 5% significance level was only exceeded for the topics dioptrics, psycho, HeartElec and VNS. Only for the topic EEG did students from the online groups achieve marginally more points (M=6.19) than the FtF_group_ (M=6.06). However, this difference is not significant.

The questions on the topic of circulation were answered significantly better by the 182 students who were taught on site than by the 16 students who were taught online (O_group_, M=7.81 vs. FtF_group_, M=9.47). 

The Spearman correlation between the number of FtF participants and the test score achieved is significant at the 0.01 level (2-sided) (p<.001). The Spearman's correlation test achieves a value of r=0.236. Therefore there is a small positive correlation, i.e. the higher the number of face-to-face attendances of a student, the more exam points were achieved in the final exam.

## Discussion

The debate about the suitability of online teaching via video conferencing as an alternative to traditional face-to-face teaching has intensified in recent years. Due to the contact restrictions in connection with the COVID-19 pandemic, educational institutions had to find new ways to maintain teaching in a very short time. In order not to exceed the permitted group size for the practical course, the Institute of Physiology at the University of Cologne offered the practical course in physiology both online via Zoom (O_group_) and “classically” on site (FtF_group_). In order to enable a largely fair distribution, the students were randomly assigned to the two different formats of the individual practical courses. With the help of the exam results and the points achieved in the individual questions, the question of whether there is a connection between the teaching format (face-to-face or online) and the performance in the final exam was investigated. The mean value comparisons of the exam points achieved in the individual questions between the online and FtF formats show that students in face-to-face classes tended to perform better than students in the online format, often even significantly better. In addition, there is a positive correlation between the number of attendances and the total number of exam points achieved. In contrast, a similar study from Thailand showed that online physiology lab instruction had the same effectiveness as the on-site lab experience [10].

One of the most important benefits of face-to-face teaching is non-verbal communication, including body language, facial expressions and eye contact, which contribute significantly to the interaction between teachers and students as well as among the students [11], [12]. A significant amount of non-verbal communication is lost during video conferencing [13]. In addition, studies have shown that students tend to participate more actively in classroom teaching and are more engaged as they are less distracted than in online environments [14], [15]. Face-to-face teaching also makes it easier to use various interactive teaching methods such as group work, discussions and spontaneous interactions, which students often find more difficult to implement in video conferencing [11]. These interactions could help to clarify misunderstandings and deepen the students’ understanding. According to a study by Freeman et al. (2007), interactive teaching can improve students’ learning and performance [6]. The opportunity to participate more actively in the classroom could also increase motivation. Intrinsic motivation and a sense of autonomy are important factors for successful learning [16]. As teachers are more aware of students’ reactions in face-to-face teaching than in a video conference, problems can be identified early on and immediate feedback can be provided. Studies by Kulik and Kulik (1988) show that immediate feedback can promote learning [17]. As students may be more affected by distractions in their environment during online lessons, this could affect their concentration and ability to focus on the subject matter. A study by Junco and Cotten (2012) shows that multitasking during learning can reduce performance [18]. Less interaction with fellow students and teachers could also have a negative impact on academic success. The study by Jaggars and Xu (2013) points to the importance of interpersonal interaction for academic success [19]. Also, online teaching requires a reliable internet connection and technical equipment. Students who do not have access to these resources or face technical challenges during online classes may thus have a disadvantage. This could be a major aspect of the issue of unequal educational opportunities [20]. Furthermore, a lack of technological infrastructure can lead to an additional burden on lecturers due to problems with technical management added to actual teaching [21], which can negatively impact on the quality of teaching.

However, there are also a number of factors that can promote the success of online teaching in the form of video conferencing, such as flexibility in terms of time and location. Online teaching via video conferencing offers a more independent learning environment for students. This could lead to students being able to use the time saved for learning approaches more effectively and individually by eliminating travelling time. According to a study by Cook et al. (2010), individualized learning approaches in medical education can help learners achieve their learning goals more efficiently [22]. According to a meta-analysis by Cavanaugh (2009), online teaching can be effective if appropriate learning strategies and supporting technologies are used [23]. Online teaching often requires a higher degree of autonomous learning from students. These skills are also of great importance in medical education because, according to a study by Kusurkar et al. (2011), self-directed learning can lead to a better understanding and application of medical knowledge [24]. In addition, online platforms enable the use of innovative teaching methods and interactive learning materials such as videos, simulations and online discussions that may not be available in face-to-face teaching. These approaches can improve the understanding of complex medical concepts. A study by Ruiz et al. (2006) shows that multimedia teaching methods can improve understanding and the persistence of aquired knowledge in medical education [25].

However, it should be emphasized that the effectiveness of online teaching depends heavily on the type of course, but also on the lecturers themselves. In our opinion, it cannot be ruled out that the quality of online teaching was influenced by the lecturers’ affinity for technology and the associated sensible use of digital media, among other things. It is also possible that the different formats influenced the observed differences in examination performance. In online classes, students did not have the opportunity to learn practical skills that come from physically interacting with equipment and materials, which could also affect students’ exam performance, even if it was not a practical exam. It should also be viewed critically that it was necessary to deviate from the original randomisation if students were unable to participate in face-to-face classes due to a (pre)-illness, e.g. a corona infection. This may well have had an influence on the poorer performance of online students. The extent to which (pre)-illnesses themselves had an influence on performance in the exam is speculative and was not analyzed. As no demographic characteristics were included in this study, dependencies on additional variables could not be adequately taken into account. Finally, it should also be noted that the study is limited to Cologne. 

## Conclusion

In the discussion of online versus face-to-face teaching in physiology practical course, our study shows that students who participated in face-to-face teaching tended to perform better in the subsequent exam questions. However, it is important to emphasize that the effectiveness of teaching depends on many variable factors (interaction, flexibility, engagement, etc.). Therefore, it is advisable to continuously review educational practices and choose the best approaches for students’ individual needs. Ideally, a hybrid solution that combines the advantages of both formats could offer an effective option.

## Authors’ ORCIDs

- Tom Dreyer: [0009-0001-6286-0140]
- Symeon Papadopoulos [0000-0002-0708-7431]
- Rudolf Wiesner: [0000-0003-1677-4476]
- Yassin Karay: [0009-0005-6380-158X]

## Competing interests

The authors declare that they have no competing interests. 

## Figures and Tables

**Table 1:**
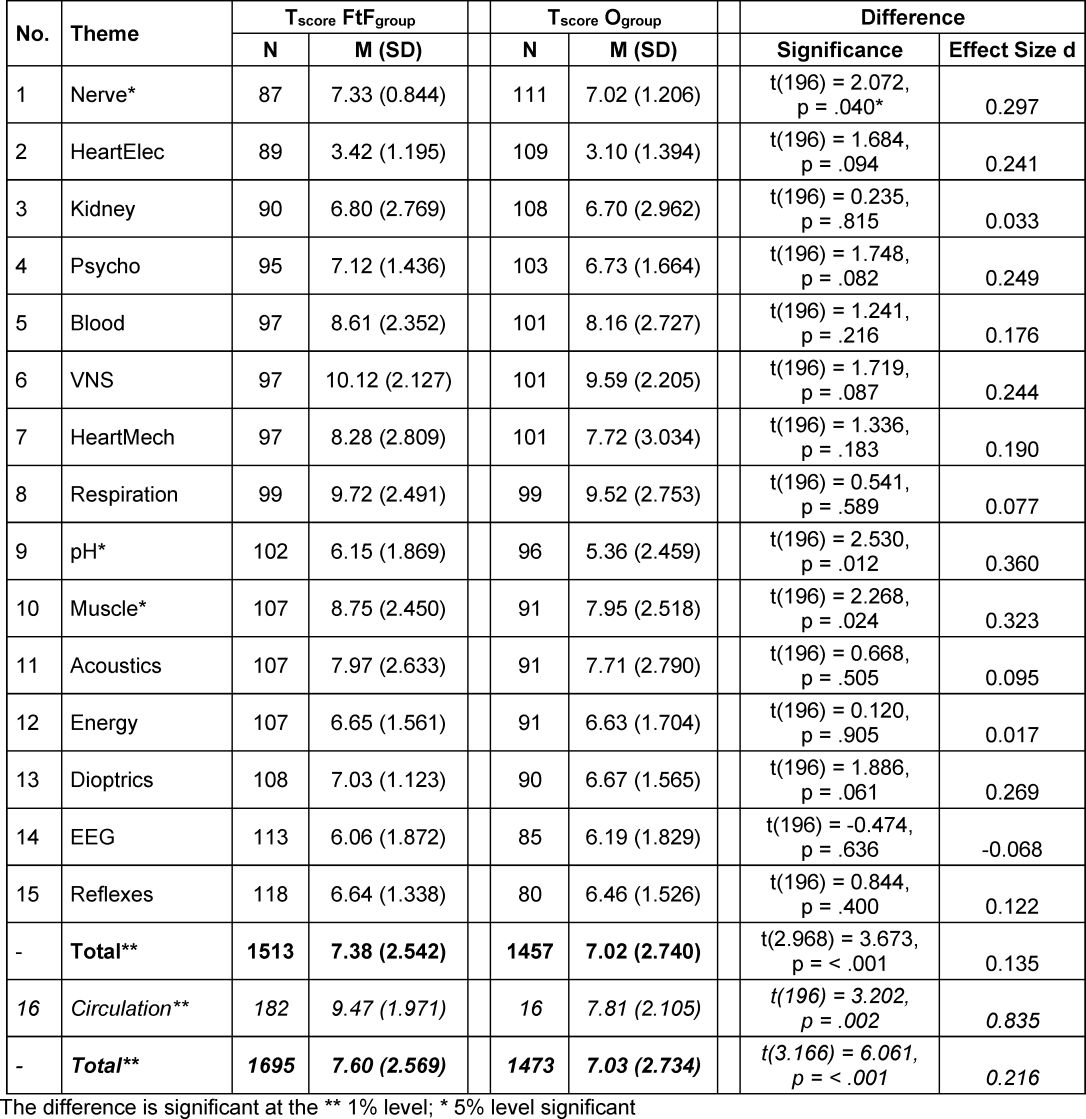
Differences and effect sizes between face-to-face and online teaching
